# The Pathophysiology and Management of Coprophagia: A Report of Two Cases and Literature Review

**DOI:** 10.1155/2018/5157879

**Published:** 2018-11-15

**Authors:** Heela Azizi, Tasmia Khan, Cecilia Canale, Jisha Kallikkadan, Ivan Leung, Alexa Kahn, Dina Rimawi, Shantale Williams, Chidubem Matthew Obegolu, Sharon Paul, Shazia Chaudhry, Chiedozie Ojimba, Kodjovi Kodjo, Olaniyi Olayinka, Oluwole Jegede, Ayodeji Jolayemi

**Affiliations:** ^1^American University of Antigua College of Medicine, Department of Psychiatry, Interfaith Medical Center, Brooklyn, NY, USA; ^2^Medical University of the Americas, Department of Psychiatry, Interfaith Medical Center, Brooklyn, NY, USA; ^3^Extern, Department of Psychiatry, Interfaith Medical Center, Brooklyn, NY, USA; ^4^Saba University School of Medicine, Department of Psychiatry, Interfaith Medical Center, Brooklyn, NY, USA; ^5^Interfaith Medical Center, Brooklyn, New York Department of Psychiatry, Interfaith Medical Center, Brooklyn, NY, USA

## Abstract

Coprophagia is a rare and distressing disorder characterized by symptoms of compulsive consumption of feces. Several attempts have been made in literature to explore the pathophysiology, management, and outcomes of this disorder. However, critical questions remain, related to characterizing effective management, features of associated comorbidities, time to recovery of symptoms, sustainability of recovery, and the determinants of outcomes. Unfortunately, there is a dearth of available literature addressing these questions. We present two cases of patients with coprophagia in seeking to address these questions. Common symptoms of thought content disorder related to coprophagic behavior are noted in both cases. A shorter time to resolution of coprophagia is noted in one of the patients compared to previously reported studies. Recent and pertinent literature was reviewed and the implications for diagnosis and management of coprophagia are discussed.

## 1. Introduction

Coprophagia is a condition described as the compulsive consumption of feces. The etymology stems from Greek origins: “copros” meaning feces and “phagein” meaning to eat. Coprophagia is well documented in literature in a number of case studies; however, the understanding of this disorder and appropriate management remains inconclusive. Several psychiatric diseases which have been associated with coprophagia include dementia [1, 2], autism [3], schizophrenia [4, 5], obsessive-compulsive disorder [1], and cognitive impairment [6]. Since these disorders do not share a common pathophysiology, the question then arises of whether there may be common features shared by these disorders that may hint at the emergence of coprophagia in such patients. Such features could include specific disorders of thought content, disturbances of perception, impulse control, or abnormalities in laboratory findings or imaging.

Given the potential for serious complications from coprophagia, a wide range of interventions both pharmacological and behavioral have been documented in literature [1, 3, 5, 7, 8]. Pharmacological classes of medications have been limited to a few second-generation antipsychotic agents and mood stabilizers. Nonpharmacological strategies are mostly based on behavioral theories [9]. The minimum time to resolution of coprophagia reported has ranged from six weeks [4] to a maximum reported time of twenty-two weeks [1]. One may also raise questions about whether we have exhausted all possible treatment interventions (clozapine or cognitive therapy) and whether we may achieve even shorter time to recovery. Such possibilities could limit the potentially serious medical sequelae of the disorder.

In seeking to address the questions raised above, we present two patients displaying coprophagic behavior. We present their comorbidities with relevant findings associated with their acute presentation with coprophagic behavior. Furthermore, we discuss specific common features of their comorbidities including psychiatric symptoms and laboratory findings. The evolution of these common features is followed while being treated with the goal being some resolution of coprophagia. If recovery is achieved, the time to recovery is noted. Of note, the time to recovery in one of the patients was a third of that reported in existing literature. Our findings pointed towards possible opportunities for pathophysiological research and management and we subsequently conducted a literature review to explore existing studies in light of our findings. The implications for future research on the pathophysiology and management of coprophagia are discussed.

## 2. Case Presentations

### 2.1. Case One

The patient reported is a 59-year-old man with a past medical history of seizure disorder and multiple inpatient psychiatric hospitalizations for manic and depressive episodes of bipolar disorder. During these prior hospitalizations, no coprophagia was reported. He was admitted to the inpatient service for an acute episode of psychotic depression with complaints of a depressed mood. He exhibited psychomotor retardation, impoverished thought content, anhedonia, and a disheveled malodorous appearance but was oriented to person, place, and time.

In the course of index hospitalization, coprophagia was noted during his first day of admission, with staff observing him defecating and consuming his feces multiple times during the day despite being placed on urgent 1:1 constant observation. Initial assessment was limited as the patient had impoverished speech and was unable to communicate possible reasons for his behavior. Laboratory work-up for possible organic etiology was only significant for a slightly low total iron binding capacity as he had an otherwise normal metabolic panel and complete blood count. Urine toxicology was negative for alcohol and illicit drugs. A brain computed tomography (CT) scan was normal showing only minimal periventricular and subcortical white matter lucencies, compatible with minimal chronic microvascular ischemic changes.

He was commenced on risperidone 2 milligrams twice daily. There was a slight improvement within the first week as he was better at communicating, but his coprophagic behavior continued unabated. The slight improvement in thought content and process enabled him to express the symptoms associated with his coprophagia. He reported command auditory hallucinations resulting in the urge to consume feces, followed by the release of guilt and tension after consumption. In addition, he expressed excessive guilt in regard to his forensic history, being a registered sexual offender, with prior incarceration for lewd conduct with three female minors. He believed his consumption of feces was a necessary retribution for his behavior, to prevent “god and society from imposing worse punishments” on him. Over the next few days on the inpatient unit, risperidone was cross-tapered with clozapine which was started at 25 mg and titrated to 125 mg per day over 12 days. Cognitive therapy was also used to explore his automatic feelings associated with his forensic history during this time period. He was compliant with both interventions. Over the course of the cross-taper and cognitive therapy, the patient reported resolution of the command hallucinations to consume feces and his excessive guilty feelings. This was accompanied by an improvement in his coprophagic behavior.

### 2.2. Case Two

The patient reported is a 32-year-old man with a past medical history of seizure disorder and a known psychiatric history of bipolar disorder with multiple inpatient hospitalizations for manic episodes during which he exhibited agitated and violent behavior. The patient has a comorbid use of synthetic cannabinoids. He was admitted to the acute inpatient service for a recurrent episode of agitation and threatening behavior in the community in the context of medication nonadherence and continuous use of synthetic cannabinoids. In the course of index hospitalization, the patient self-reported his coprophagic behavior during his initial psychiatric assessment. The patient stated that he believed that the consumption of feces was preventing him from going to jail and facing the death penalty for a murder that he claimed he committed in the past. He was unable to describe any specifics for his murder but felt that he deserved unusually cruel and harsh punishment from law enforcement that could only be prevented by consuming feces. This feeling of guilt and continued belief that he was deserving of cruel and harsh punishment induced his coprophagic behavior. He did not report any associated command auditory hallucinations but expressed depressed mood as he discussed his reasons for feeling guilty. His affect was however incongruent with his stated depressed mood. He was oriented to person, place, and time. Laboratory work-up was within normal limits and a urine toxicology panel (which does not detect synthetic cannabinoids) was also negative for illicit substances. He declined brain imaging for unclear reasons.

The patient was started on a course of risperidone 2 mg twice daily, by mouth, and divalproex sodium 750 mg orally twice a day for mood stabilization. He continued to display mood instability with multiple periods of agitation and aggression, as well as coprophagic behavior. His expressed reasons for coprophagia remained the same. He declined any psychotherapeutic interventions to address his excessive guilt and automatic negative cognitions. Over the course of a few days, risperidone was titrated to 6 mg daily in divided doses, with augmentation of his treatment with 200 mg every eight hours orally for mood stabilization/impulsivity in addition to divalproex sodium. The patient's mood symptoms improved after a few days on the current regimen; he became less irritable and easily redirectable, exhibited less impulsivity, and expressed more logical thinking process. His activities of daily living also improved, as he was less malodorous and exhibited improved grooming and hygiene. The patient's coprophagic behavior, however, did not resolve. He continued to express the same guilt and desire of unusually cruel and harsh punishments for his previous transgressions. He was discharged on the twenty-sixth day of admission with resolution of his acute manic episode. His coprophagic behavior and feelings of guilt did not show any resolution.

## 3. Discussion

The two patients discussed in this report present with symptoms of coprophagia in the context of an acute exacerbation of chronic psychiatric disorders. In prior case reports in the literature, the disorders often associated with coprophagia were cognitive disorders [1, 2] and schizophrenia spectrum disorders [4, 5]. Of note, these patients presented with affective spectrum disorders (bipolar disorder). No significant neurocognitive disorders were seen in either patient. The only neurological disorder observed in both patients was seizure disorder. These cases seem to raise further questions of common pathophysiology of coprophagia with the expansion of associated comorbidities. Further examination of their clinical presentation revealed similarities in symptoms that may need to be explored in a literature review and future studies. For instance, both patients expressed seemingly excessive guilt and believed they were deserving of serious punishment for past misdeeds, which could only be prevented by coprophagic behavior. It is unclear if the feelings of guilt achieved levels of delusional thought content in both patients, but it was pervasive and severe enough to lead to coprophagic behavior. One of the patients had congruent command hallucinations to act out such self-imposed punishment. A question may arise of whether such cognitive distortions are only associated with coprophagia that occurs in the context of affective disorders or whether disorders of thought content or perception can be seen in coprophagia associated with cognitive and schizophrenia spectrum disorders.

The medical comorbidities in this case series did not provide possible directions on underlying pathophysiology as much as the psychiatric presentations. While both patients had a previous known medical history of seizure disorder, neither exhibited a seizure episode during the admission. In addition, the finding of a low total iron binding capacity was not seen in the second patient. A comparison of possible associated findings on imaging could not be accomplished as patient two did not consent to neuroimaging. The findings on the first patient, however, were mostly suggestive of vascular disease.

The management of both patients was also notable in terms of their nature and outcomes. Both patients had been given risperidone. The second patient had a higher total risperidone dose than the first patient. He was also managed in combination with two mood stabilizers for a longer time period, for a total of twenty-six days without any resolution of the coprophagia. The first patient achieved recovery of coprophagia after 12 days of augmentation of risperidone with clozapine with a total period of treatment of 19 days. This is a significantly shorter period than the six-week recovery reported in literature and shorter than the eight weeks reported on aripiprazole. Of note, the resolution of coprophagia was associated with the resolution of excessive guilt and cognitive distortions related to deserving punishment. These symptoms never resolved in the second patient, despite mood stabilization. These may further motivate the case of cognitive distortions as underlying the symptoms of coprophagia.

As a result of these questions on pathophysiology and management of coprophagia, we conducted a literature review on pharmacological approaches to coprophagia with a goal of determining the possible benefits of the psychotropics in both cases presented in this article. We aimed to identify peer-reviewed articles related to the etiology of coprophagic behavior. We searched PubMed for articles on the cause of coprophagia without restriction to time-period or language. The search was conducted using the keywords and MeSH terms: “etiology or aetiology and coprophagia.” We also searched the reference list of eligible articles to identify additional articles relevant to this study. Endnote was used for citation management and de-duplication of references. Screening for eligible articles was conducted independently by five authors. Eligible studies were those that focused on the cause or risk factors for coprophagic behavior in human subjects.

Given the paucity of articles on this topic, all types of studies were considered for analysis including experimental, cohort, case-control, case series, and case reports. Studies conducted in animals and those in which a clear etiology was not stated were excluded. Although there were multiple articles that reported on the same study population, only the most recent publication was included in our final review. Any disagreement regarding the eligibility of an article was resolved by discussion among the authors. Relevant data from eligible articles were extracted and entered into a data abstraction form designed by the authors using Microsoft Excel. Data extracted from eligible articles include last name of first author, publication year, type of study, characteristics of study population, psychiatric assessment of coprophagia, treatment offered and specific duration, treatment outcome, and conclusion.

A summary of the cases extracted is shown in Tables 1 and 2.  Table 1 covers the pathophysiology by exploring comorbidities and concurrent symptoms while Table 2 covers management and outcomes. All the studies reported the gender of patients [1, 3, 4, 7–10]. Forty percent were male and the age range of subjects was 7 years to 94 years; the mean age was 52.25 years.

In terms of associated comorbidities and pathophysiology, patients with coprophagia presented with co-occurring illnesses, such as developmental delay [5], intellectual disability [6], aging disorders (dementia and Alzheimer's disease) [2], depression [10], schizoaffective disorder [5], epilepsy [1], and brain tumors [1]. Patients presented with co-occurring symptoms, such as incontinence, fecal smearing, [6], abnormal mouth movements, episodically aggressive behavior, wandering, speech disturbances [15], and hypersexuality [1]. Neuroimaging findings were diverse in the literature review, with many of the findings dependent on the underlying neurological disorders [1]. In some patients, imaging studies reported cerebral atrophy, particularly of the medial temporal lobe [1]. As found in our case series, the laboratory findings were diverse [2–4]. We also explored psychological symptoms which were also noted to be diverse. Only one case report [10] reported similar symptoms of shame and self-disgust as seen in our cases, with ours being the second and third. The case reported by Joseph et al [10] also had an affective disorder (depressive disorder) and a substance use disorder. This may lead to the exploring coprophagia as a symptom associated with underlying disorders of a thought content especially in patients with affective disorders.

The literature review also showed that multiple approaches were used for treatment such as medications [5], antipsychotics, [3, 8], and anticonvulsants [7]. In particular, first-generation antipsychotics such as haloperidol [1] and atypical antipsychotics such as ziprasidone [8], aripiprazole [3], and quetiapine were documented for coprophagic relief secondary to psychosis. Patients were also placed on multiple medication regimens. One patient was given multiple drugs (donepezil 10 mg daily, memantine 10 mg daily, ziprasidone 40 mg at bedtime, duloxetine 90 mg daily, and mirtazapine 15 mg daily) and carbamazepine regimen titrated to a maximum dose of 300 mg three times daily [8]. There were no reported cases of the use of clozapine.

In terms of time to recovery, only five articles reported the time it took for the coprophagic behavior to respond (six weeks [4], eight weeks [3], eight months [9], and twenty-two months [1]). On average, the coprophagic behavior resolved or improved after one month. The time to recovery did not seem to be dependent on the number of concurrent medications used, as the lowest time to recovery achieved on medications as reported in the literature was with aripiprazole only. While time to recovery on some anticonvulsants [7] and antidepressants [1, 4] was not clear in the review, the characterization of the outcomes of the patients did not clearly point to recovery, as one of the studies reported improvement only when antipsychotics were added. This is consistent with our findings in the second patient who seemingly achieved no benefits with the anticonvulsant augmentation. The time to recovery of our first patient with clozapine augmentation (12 days) is shorter than the shortest time to recovery reported in the literature review (six weeks) [4]. The role of clozapine as a preferred medication for coprophagia may need to be studied further. None of the case studies reported the resolution of psychiatric symptoms (guilt, shame, and feelings of tensions) and thus it remains unclear if the resolution of coprophagia was secondary to treatment of underlying psychiatric disorders. Our case series motivates an exploration of coprophagia as occurring in the context of thought content disorders in underlying psychiatric disorders.

## 4. Conclusion

The pathophysiology and management of coprophagia remains a subject of interest with a number of questions that need to be answered. The diversity of associated psychiatric comorbidities may point towards the need to explore common psychological symptoms, such as thought content disorders, that lead to the emergence of coprophagia. Future studies on pathophysiology may focus on commonalities of specific thought, perceptual, or affective disorders rather than the identification of diagnostic comorbidities in determining the pathophysiology. The management of coprophagia may also be further optimized by choice of medications such as clozapine. Future studies may be needed to compare medications such as clozapine with other antipsychotics, with outcome measures that include degree of recovery and time to recovery.

## Figures and Tables

**Table 1:** Review of selected literature on the comorbidities and concurrent symptoms of coprophagia.

**Article **	**Gender**	**Age**	**Illnesses**	**Symptoms and Signs**	**Lab Findings/Imaginig Studies**
Coprophagia in an 8-Year-Old Hospitalized Patient: A Case Report and Review of the Literature. Bacewicz, et al., 2017. [9]	Male	8 years old	Feculent emesis of well-formed stool	Oral fixation, immaturity, denied coprophagia	

Coprophagia in an elderly man: a case report and review of the literature. Beck, et al., 2005. [5]	Male	77 years old	Mild mental retardation, cognitive dysfunction, depression		

Coprophagic cafè coronary. Byard, et al., 2001. [11]	Male	74 years old	Multiinfarct dementia and atherosclerosis	Found dead attributed to upper airway obstruction due to a bolus of fecal material impacted within the laryngopharynx	

A case of coprophagia presenting with sialadenitis. Donnellan, et al., 1999. [12]	Female	94 years old	Dementia and recurrent submandibular sialadenitis secondary to coprophagia	Disoriented, incontinent	Tomography scan of brain: generalized cerebral atrophy

Coprophagic Asphyxation in an Intellectually Disabled Woman. Erickson, et al., 2017. [13]	Female	36 years old	Intellectual disability, bipolar disorder, schizoaffective disorder, aging disorders	Found dead due to aspiration of stool bolus during an episode of coprophagia	No signs of vitamin, iron, or thiamine deficiency, no abnormal GI and brain findings

Treatment of a retarded child's faeces smearing and coprophagic behaviour. Friedin, et al., 1979. [14]	Male	7 years old	Intellectual disability	Incontinence, smearing his own feces, coprophagia	

A clinical study of adult coprophagics. Ghaziuddin, et al., 1985. [15]	14 patients (2 Male and 12 Female)	Average age was 71. The youngest patient was 61 years old	Epilepsy (2), depression, cerebral atrophy and left hemispheric cognitive dysfunction (1), fluctuating topic confusional states (2)	Speech disturbances (10), wanderers (8), persistent abnormal mouth movements (4), episodically aggressive (6)	Normal thiamine levels

Coprophagia in neurologic disorders. Josephs, et al., 2016. [1]	12 patients (6 Male and 6 Female)	Average age was 55 (20-88 years)	Neurodegenerative dementia (6), developmental delay(2) seizures (1), steroid psychosis(1), frontal lobe tumor(1), schizoaffective disorder (1)	Fecal smearing (6), aggression (5), hypersexuality(4), pica (4)	Moderate-to-severe medial temporal lobe atrophy and frontal lobe atrophy (6), brain imagining associated with the patient diagnosis (4), brain imaging normal (2)

Coprophagia and urodipsia in a chronic mentally ill woman. McGee, et al., 1989. [4]	Female	Not reported (old)	Residual schizophrenia and depression	Urodipsia	

Aripripazole treatment for coprophagia in autistic disorder. Pardini, et al., 2010. [3]	Male	29 years old	High functioning autistic spectrum disorder		Blood studies (including thiamine concentration) and neurological examination were unrevealing

Coprophagia and pica in individuals with mild to moderate dementia and mixed (iron deficiency and microcytic) anemia. Sharma, et al., 2011. [2]	Female	83 years old	Alzheimer's disease, major depressive disorder, and mixed (iron deficiency and microcytic) anemia		

Treatment of coprophagia with carbamazepine. Stewart, 1995. [7]	Male	46 years old	Left frontotemporal multiform glioblastoma	Severe dementia, global aphasia, hemiparesis, seizure disorder	

Escalation of a fetish: coprophagia in a nonpsychotic adult of normal intelligence. Wise, et al., 1995. [10]	Male	47 years old	Depression and alcohol abuse	Coprophilia, shame, self-disgust	

**Table 2:** Review of selected literature on the management and outcomes of coprophagia.

**Article **	**Treatment**	**Outcome of Treatment**	**How long for the coprophagic behavior to respond**
Coprophagia in an 8-Year-Old Hospitalized Patient: A Case Report and Review of the Literature. Bacewicz, et al., 2017. [9]	1:1 observation and psychiatry consult. Outpatient psychiatric services after dismission	Coprophagic behavior ceased	Immediately after the 1:1 observation

Coprophagia in an elderly man: a case report and review of the literature. Beck, et al., 2005. [5]	Sertraline 25 mg daily	Coprophagic behavior ceased	

Coprophagic cafè coronary. Byard, et al., 2001. [11]	Not applicable	Not applicable	Not applicable

A case of coprophagia presenting with sialadenitis. Donnellan, et al., 1999. [12]	Antibiotics	Coprophagic behavior ceased	

Coprophagic Asphyxation in an Intellectually Disabled Woman. Erickson, et al., 2017. [13]	Not applicable	Not applicable	Not applicable

Treatment of a retarded child's faeces smearing and coprophagic behaviour. Friedin, et al., 1979. [14]	Toilet training	Coprophagic behavior reduced	8 months

A clinical study of adult coprophagics. Ghaziuddin, et al., 1985. [15]	Not discussed	Not discussed	Not discussed

Coprophagia in neurologic disorders. Josephs, et al., 2016. [1]	Behavioral treatment (mittens and behavioral modification technique), Haloperidol, Lorazepam, Citalopram, Trazodone, Mirtazapine, Valproic Acid, Quetiapine	Coprophagic behavior ceased only when used Haloperidol was used (1-3 mg daily)	

Coprophagia and urodipsia in a chronic mentally ill woman. McGee, et al., 1989. [4]	Reproductive and rewarding activities (sewing, art group, music appreciation group)	Coprophagic behavior ceased	6 weeks

Aripripazole treatment for coprophagia in autistic disorder. Pardini, et al., 2010. [3]	Aripripazole (15 mg daily)	Coprophagic behavior reduced substantially	8 weeks

Coprophagia and pica in individuals with mild to moderate dementia and mixed (iron deficiency and microcytic) anemia. Sharma, et al., 2011. [2]	behavioral changes, including antecedent manipulation, discrimination training between edible and nonedible items, self-protection devices that prohibit placement of objects in the mouth, sensory reinforcement, differential reinforcement of incompatible behaviors such as screening (covering eyes briefly), contingent aversive oral taste (lemon), contingent aversive smell sensation (ammonia), contingent aversive physical sensation (water mist), brief physical restraint, and overcorrection (correct the environment or practice appropriate alternative responses). Donepezil 10 mg daily, Memantine 5 mg twice daily, Ziprasidone 40 mg at bedtime, Duloxetine 90 mg daily, and Mirtazapine 15 mg daily (medication that was administered before the coprophagic behavior)	Patient improved	

Treatment of coprophagia with carbamazepine. Stewart, 1995. [7]	Carbamazepine regimen titrated to a maximum dose of 300 mg tid, Phenytoin 100 mg bid, Dexamethasone 2 mg tid, Lorazepam	Coprophagic behavior ceased only with Carbamazepine	

Escalation of a fetish: coprophagia in a nonpsychotic adult of normal intelligence. Wise, et al., 1995. [10]	Supportive psychotherapy and tricyclic antidepressant, alcoholics anonymous meetings	Coprophagic behavior ceased	22 months
